# PET2 response associated with survival in newly diagnosed diffuse large B-cell lymphoma: results of two independent prospective cohorts

**DOI:** 10.1038/s41408-022-00649-x

**Published:** 2022-05-03

**Authors:** Sanjal H. Desai, Levi Pederson, Betsy LaPlant, Raphael Mwangi, Matthew Maurer, Jason R. Young, William R. Macon, Rebecca L. King, Yucai Wang, James R. Cerhan, Andrew Feldman, David J. Inwards, Ivana Micallef, Patrick Johnston, Luis F. Porrata, Stephen M. Ansell, Thomas M. Habermann, Thomas E. Witzig, Grzegorz S. Nowakowski

**Affiliations:** 1 Division of Hematology, Department of Internal Medicine, Mayo Clinic, Rochester, MN USA; 2 Department of Quantitative Health Sciences, Mayo Clinic, Rochester, MN USA; 3 Division of Nuclear Medicine, Department of Radiology, Mayo Clinic, Jacksonville, FL USA; 4 Department of Laboratory Medicine and Pathology, Mayo Clinic, Rochester, MN USA

**Keywords:** Cancer, Lymphoma

## Abstract

Studies evaluating Positron Emission Tomography scan after 2 cycles of chemotherapy (PET2) in newly diagnosed diffuse large B cell lymphoma (DLBCL) are heterogeneous in patient characteristics, treatments and have conflicting results. Here we report association of PET2 with outcomes in two large independent prospective cohorts of newly diagnosed DLBCL pts treated with two RCHOP-based regimens. The discovery cohort consisted of pts enrolled in single arm phase 2 MC078E study of lenalidomide with RCHOP (R2CHOP). The validation cohort consisted of RCHOP-treated pts from the Molecular Epidemiology Resource (MER) cohort. Pts who received 3-6 cycles of therapy and had PET2 were included in the study. Patients who progressed on PET2 were excluded. Revised response criteria 2007 were used to define PET2 response PET2 positive (PET2 + ) pts had inferior EFS [24-month EFS 45.5% vs 87.9%, HR 4.0, CI_95_ (2.1–7.9), *p* < 0.0001) with a trend towards lower OS [24-months OS 77% vs 94.8%, HR 2.0, CI_95_ (0.9–4.8), *P* = 0.1] than PET2 negative (PET2−) pts in MC078E cohort. PET2 + pts had an inferior EFS (24 month EFS 48.7% vs 81.6%, HR 2.9, CI_95_ 2.0–4.2, *p* < 0.0001) and OS (24-month OS 68.6% vs 88.1%, HR 2.3, CI_95_: 1.5–3.5, *p* < 0.0001) in the MER cohort. These results were consistent regardless of age, sex and in the subgroup of advanced stage and high-risk international prognostic index (IPI). For MER, PET2 + pts also had higher odds of positive end of treatment PET (OR: 17.3 (CI_95_ 7.9–37.7), *p* < 0.001). PET2 is an early predictor DLBCL pts at high risk of progression and death in two independent prospective cohorts. PET2-guided risk-adapted strategies may improve outcomes, and should be explored in clinical trials.

## Introduction

Although, salvage therapy and autologous stem cell transplant (ASCT) may cure up to 40% of diffuse large B cell lymphoma (DLBCL) patients who relapse on frontline chemoimmunotherapy, patients who progress after ASCT or have chemorefractory relapse have poor outcomes [1–4]. Moreover, patients with primary refractory disease or early relapse have poor response rates of 26% to further lines of therapy and median overall survival (OS) of 6.3 months [4]. Identifying biomarkers for risk of relapse is an area of investigative need and when identified may facilitate development of risk-adapted therapies that can potentially improve outcomes.

Response to interim F^18^ flurodeoxyglucose positron emission tomography/computed tomography (PETCT) during frontline chemoimmunotherapy has been explored as a prognostic marker in newly diagnosed DLBCL in multiple studies with small sample size, heterogeneous timing of interim PETCT, heterogeneous populations, variable study treatments and conflicting results [5–12]. Three major prospective studies have evaluated impact of PETCT after cycles 2 of frontline chemoimmunotherapy (PET2) on outcomes of newly diagnosed DLBCL with conflicting results [8–10]. Positive PET2 defined as Deauville score (DS) of 3,4 or 5 was associated with inferior progression free survival (PFS) but not overall survival (OS) in patients treated with rituximab, cyclophosphamide, doxorubicin, vincristine, and prednisone (RCHOP) every 14 days (RCHOP-14) [8, 13]. An analysis of CALGB 50303 trial did not find significant associations of PET2 response by Deauville Score (DS) with survival in patients treated with standard RCHOP or dose escalated etoposide, prednisone, vincristine, cyclophosphamide, doxorubicin, and rituximab (DA-EPOCH-R) [10]. In the UK National Cancer Research Institute Prospective Study of patients treated with standard RCHOP or RCHOP-14, DS of 5 predicted worse PFS and OS. This study population consisted of patients treated with either standard RCHOP or RCHOP-14 [9].

Here we explore association of PET2 response with outcomes of newly diagnosed DLBCL in two large independent, prospective cohorts treated homogenously with two standard RCHOP-based regimens proven to have similar efficacy in randomized controlled clinical trials [14, 15]. The discovery cohort consists of patients enrolled in single arm phase 2 MC078E study that evaluated RCHOP with lenalidomide (R2CHOP) in newly diagnosed DLBCL. The validation cohort consisted of newly diagnosed DLBCL patients who received RCHOP and were enrolled in Molecular Epidemiology Resource (MER) cohort at the Mayo Clinic [16].

## Patients and methods

### Discovery cohort

Discovery was conducted using MC078E cohort which comprised of adult patients with newly diagnosed DLBCL who were enrolled into single arm phase 2 MC078E study [17]. Study eligibility criteria, procedures and treatments have been previously described [17]. All enrolled patients received lenalidomide 25 mg (day 1–10) with standard doses of rituximab (day 1), cyclophosphamide (day 1), vincristine (day 1), doxorubicin (day 1) and prednisone (day 1–5) (R2CHOP) every 21 days for up to 6 cycles. All patients received pegfilgrastim 6 mg subcutaneous on day 2 of 21-day cycle. All patients enrolled in MC078E trial underwent PETCT at baseline, in the interim (after 2 or 3 cycles) and 1–3 weeks after end of treatment (EOT). In this study PET was reviewed centrally according to Revised Response Criteria for Malignant Lymphoma (2007 version) [18]. Patients who had interim PETCT before cycle 3 (PET2) were included in the discovery cohort. Patients who progressed on PET2 were excluded from the discovery cohort. Positive PET2 was defined as focal or diffuse increase in activity above background at a site that is incompatible with normal anatomy or physiology [18].

The study was conducted according to declaration of Helsinki and was approved by Mayo Clinic institutional review board (IRB) (protocol MC078E). MC078E study was registered at clinicalTrial.gov. (NCT00670358)

### Validation cohort

Validation was performed using adult patients with newly diagnosed DLBCL enrolled at Mayo Clinic from the prospective observational Molecular Epidemiology Resource (MER) cohort of Mayo Clinic Rochester and University of Iowa SPORE; details of this cohort have been previously described [16]. Patients with lymphoma who were within 9 months from their initial diagnosis at presentation were enrolled into the MER from 1 September 2002 to 30 June 2015. All participants provided written informed consent, and the cohort protocol was approved by the institutional review boards at the Mayo Clinic and the University of Iowa. All participants were treated according to treating physician’s choice and were systematically contacted every 6 months (±4 weeks) from the date of original diagnosis for the first 3 years and then annually thereafter for follow up. Follow-up data include disease recurrence or progression after frontline treatment and vital status. All the events were verified through medical record review. For validation cohort, we queried MER database in January 2021.

All consecutive cases of newly diagnosed DLBCL who were treated with 3-6, 21-day cycles of standard doses of rituximab, cyclophosphamide, vincristine, doxorubicin, and prednisone (RCHOP) and had baseline PET as well as PET2 were eligible. Patients who progressed on PET2 were excluded from this study. Demographic (age, sex) and clinical (stage, extra nodal involvement, international prognostic index (IPI), performance status) characteristics were recorded at baseline. PET2 and EOT PET were clinically reviewed by treating radiologists and results were uploaded in electronic health records and were abstracted by retrospective review. PET positivity/negativity threshold was determined by Revised Response Criteria for Malignant Lymphoma (2007). Positive PET2 was defined as focal or diffuse increase in activity above background at a site that is incompatible with normal anatomy or physiology [18].

### Outcomes

Primary outcome was event free survival (EFS) by PET2 status. EFS was defined as time from date of PET2 to progression, new unplanned lymphoma-directed treatment, or death from any cause. Secondary outcomes were overall survival (OS) by PET2 status and association of PET2 status with EOT PET status (positive or negative). OS was defined by time from PET2 to death or last follow up. Analysis of EFS and OS by PET2 status was conducted within subgroups defined by age, sex, IPI and stage.

### Statistical analysis

Continuous and categorical data were summarized with descriptive statistics. Chi-squared test and Fisher’s exact test was used to evaluate differences between categorical variables. Differences in EFS and OS by PET2 status were evaluated using Kaplan-Meier curves and Cox proportional hazards models. EFS and OS by PET2 status were evaluated in the subgroups of age, sex, IPI and stage using univariate COX proportional hazard model. Time to event endpoints were calculated from time of PET2. Statistical analyses were conducted using SAS version 9.4 and *p* < 0.05 was considered significant. Data met the assumptions of statistical tests and variance were similar between groups compared.

## Results

### MC078E cohort (discovery)

**Fig. 1:**
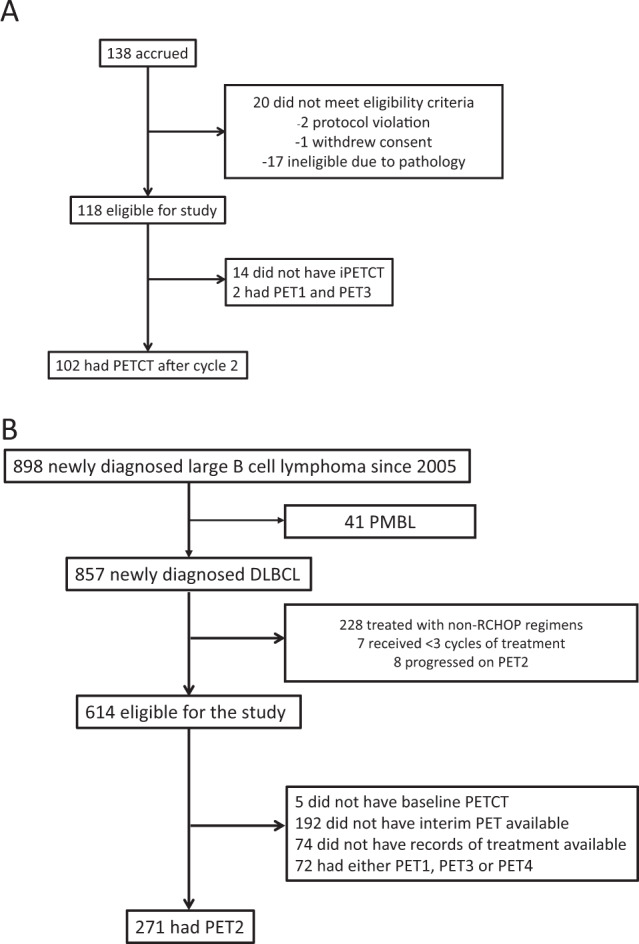
CONSORT Diagram. **A** MC078E cohort. **B** MER cohort. Abbreviations. MER; molecular epidemiology resource; PETCT; Positron Emission Tomography computed tomography, iPETCT; interim PETCT, PET1, PET2 and PET3; PETCT scan after first, second and third cycle respectively, PMBL; primary mediastinal B cell lymphoma, DLBCL; diffuse large B cell lymphoma, RCHOP; rituximab, cyclophosphamide, doxorubicin, vincristine and prednisone every 21 days, MR-CHOP; RCHOP with intravenous methotrexate.

**Fig. 2:**
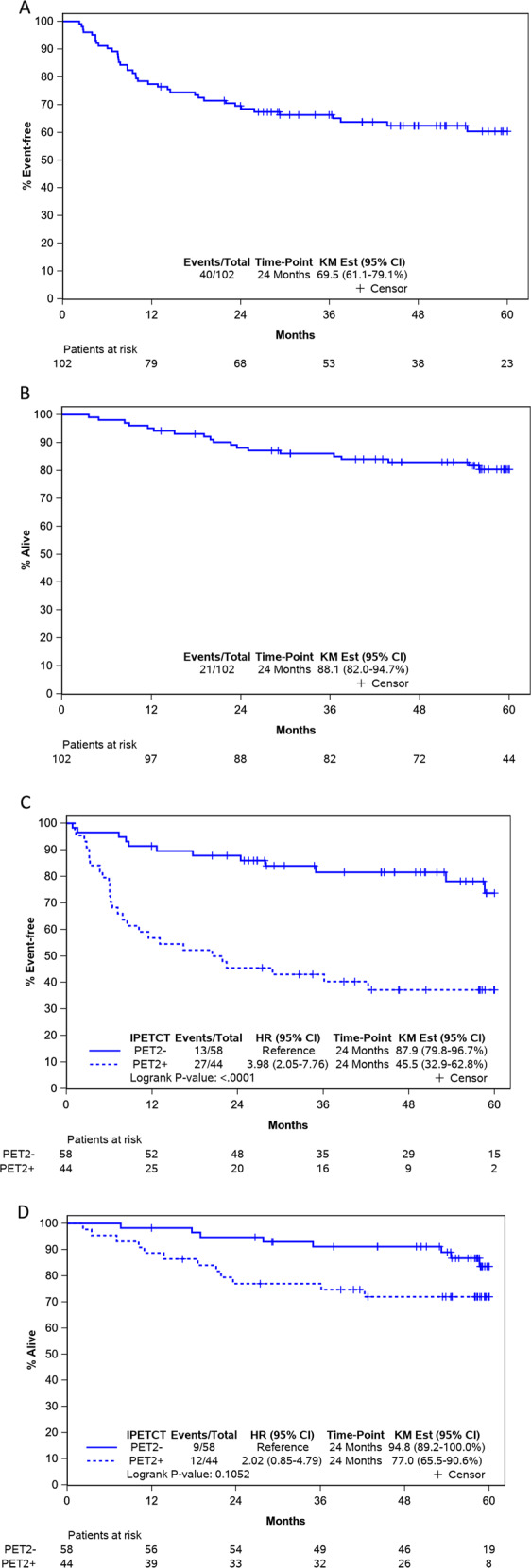
Survival in discovery cohort. **A** MC078E: event free survival (EFS). **B** MC078E: overall survival (OS). **C** MC078E: event free survival (EFS) by PET2 from time of PET2. **D** MC078E: overall survival (OS) by PET2 from time of PET2. Abbreviations. PET2; Positron Emission Tomography scan after cycle 2, CI_95_; 95% confidence interval.

Of 118 patients who were enrolled in the trial and 102 with PET2 were included in the discovery cohort (Fig. 1A). Table 1 presents baseline characteristics of MC078E cohort. Median age was 64 (19–87) years. Sixty-one (60%) were male, 89 (87%) had advanced stage, 46 (45%) had high IPI and 64 (63%) had extra nodal involvement. Median number of extra nodal sites were 1 (range: 0–8). All patients received R2CHOP. Ninety-four (92%) completed 6 cycles and all received at least 3 cycles of therapy. Median follow-up was 60 months (range: 13–83). Fifty-eight patients were PET2 negative (PET2−) and 44 were PET2 positive (PET2 + ), Kaplan–Meier estimates of 24-month EFS and OS from PET2 scan for the 102 patients with PET2 evaluation were 69.5% (CI_95_ 61.1–79.1) and 88.1% (CI95 82.0–94.7), respectively (Fig. 2A & 2B, respectively). PET2 + patients had significantly lower EFS [24-month EFS 45.5 vs 87.9%, HR 4.0, CI_95_ (2.1–7.8), *p* < 0.0001) and a trend towards lower OS [24-month OS 77% vs 94.8%, HR 2.0, CI_95_ (0.9–4.8), *P* = 0.1] (Fig. 2C, D, respectively).

**Table 1:** MC078E: patient baseline characteristics.

Patient characteristics	Total (*N* = 102)
Age, median (range)	64 (19–87)
Age > 60, *n* (%)	60 (59)
Sex, *n* (%)	
Female	41 (40)
Male	61 (60)
Performance score, *n* (%)	
0	57 (56)
1	38 (37)
2	7 (7)
Stage, *n* (%)	
II	13 (13)
III	31 (30)
IV	58 (57)
Number of EN sites	
Median (range)	1.0 (0–8)
EN involvement, *n* (%)	64 (63)
IPI Group, *n* (%)	
0–2	56 (55)
3–5	46 (45)
PET2 result, *n* (%)	
Negative	58 (57)
Positive	44 (43)

*N* number of patients, % percentages, *EN* extra nodal involvement, *IPI* international prognostic index, *PET2* Positron Emission Tomography after cycle 2.

Ninety-eight patients had EOT PET available, 83 (85%) were PET− and 15 (15%) were PET + . Out of 44 with PET2 + 42 had EOT PET available and 15 remained positive at EOT PET. Out of 58 PET2−, 56 had EOT PET available and all were PET−. Since all PET2− patients were EOT PET−, the association of PET2 status with EOT PET status was not analyzed.

### MER cohort (validation)

Out of 857 DLBCL pts enrolled into MER between August 2005 and June 2015 at the Mayo Clinic, 614 were treated with RCHOP for 3-6 cycles and 271 had available PET2 (MER) (Fig. 1B). Table 2 lists baseline characteristics of MER cohort. There was no difference in baseline characteristics of patients with and without PET2 (Table 1). In the study population of 271 patients, median age was 65 years (range 55–73), 151 (56%) were male, 173 (64%) had advanced stage, 103 (38%) had IPI 3–5. One hundred eighty-five (68%) were PET2− and 86 (32%) were PET2 + .

**Table 2:** MER: Patient Baseline Characteristics.

Characteristics	PET2 (*N* = 271)	No PET2 (*N* = 343)	*P* value
Age, median (range)	65 (18–89)	63 (18–93)	0.6
Age >60, *n* (%)	170 (63)	206 (60)	
Gender, *n* (%)			
Female	120 (44)	140 (41)	0.5
Male	151 (56)	203 (59)	
Performance score, *n* (%)			
<2	242 (89)	310 (91)	0.5
>=2	29 (11)	31 (9)	
Missing	0	2	
No. of EN sites, *n* (%)			
<=1	205 (76)	277 (81)	0.1
>1	66 (24)	65 (19)	
Ann Arbor Stage Group, n (%)			
I–II	98 (36)	148 (43)	0.07
III–IV	173 (64)	194 (57)	
Missing	0	1	
IPI Group, *n* (%)			
0–2 Low	168 (62)	231 (67)	0.2
3–5	103 (38)	112 (33)	
PET2 result			
Positive	86 (32)	n/a	
Negative	185 (68)	n/a	

*N* number of patients, % percentages, *EN* extra nodal involvement, *IPI* international prognostic index, *PET2* Positron Emission Tomography after cycle 2, *n/a* not applicable.

**Fig. 3:**
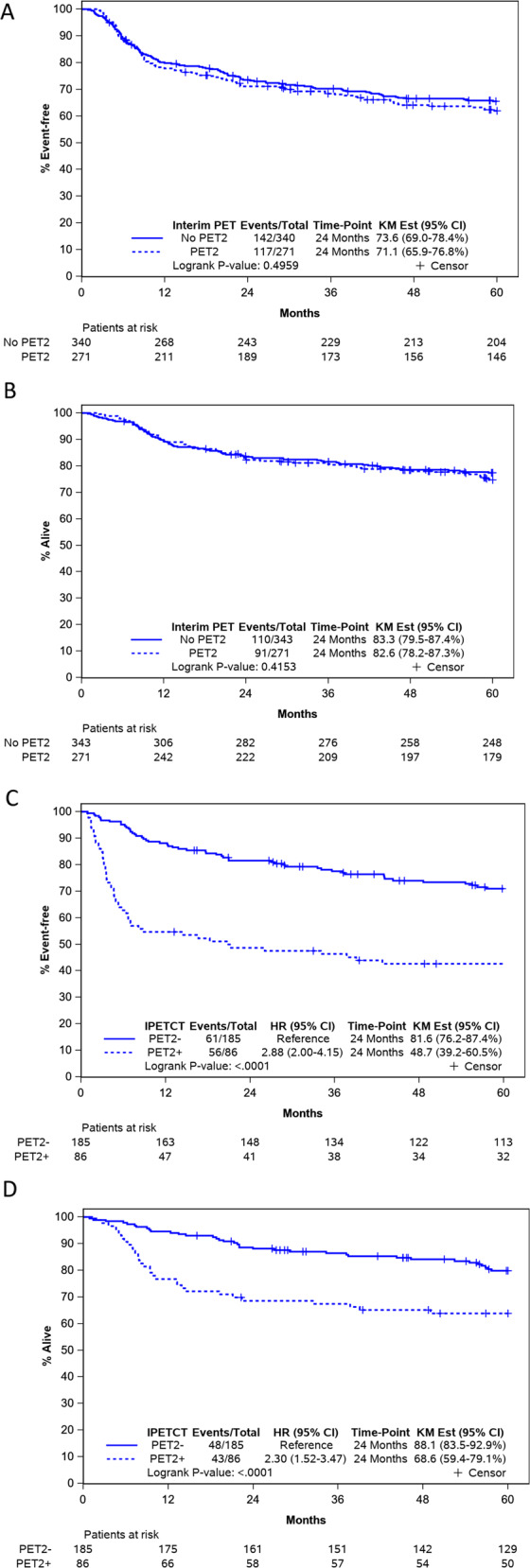
Survival in MER cohort. **A** MER: event free survival (EFS). **B** MER: overall survival (OS). **C** MER: event free survival (EFS) by PET2 from time of PET2. **D** MER: overall survival (OS) by PET2 from time of PET2. Abbreviations. PET2; Positron Emission Tomography scan after cycle 2, HR; hazard ratio, CI_95_; 95% confidence interval, MER; Molecular Epidemiology Resource cohort, PET2; Positron Emission Tomography scan after cycle 2, HR; hazard ratio, CI_95_; 95% confidence interval.

Median follow up was 95 (range: 18–180) months. Kaplan–Meier estimates of 24-month EFS and OS were 71.1% (CI_95_ 65.9–76.8) and 82.6% (CI_95_ 78.2–87.3) (Fig. 3A, B, respectively). There was no significant difference between EFS and OS of patients with and without PET2. Compared to PET2−, PET2 + pts had significantly inferior EFS (24-month 48.7% vs 81.6%, HR 2.9, CI_95_ 2.0–4.2, *p* < 0.0001, Fig. 3C) and also had significantly lower OS (24-month 68.6% vs 88.1%, HR 2.3, CI_95_: 1.5–3.5, *p* < 0.0001, Fig. 3D).

Of 234 patients with both PET2 and EOT PET available, 181 (77%) had negative EOT PET and 53 (23%) had positive EOT PET. PET2 + pts had higher odds of positive EOT PET (OR: 17.3 (CI_95_ 7.9–37.7), *p* < 0.001) and progression on EOT PET (OR: 4.3 (CI_95_ 1.9–9.8), *p* < 0.001) compared to PET2- patients in MER cohort.

### Subgroup analysis

Next, we assessed univariate association of PET2 response with EFS and OS in the subgroups stratified by age, gender, stage and IPI. For these analyses, we combined patients of discovery and validation cohort (Supplementary Fig. 1A & 1B). Negative PET2 was consistently associated with superior EFS but not OS regardless of age and gender. PET2 response was associated with significantly higher EFS and OS in the subgroup of IPI 3-5 and advanced stage, but not in low risk or early stage disease (Supplementary Fig. 1A & 1B).

Supplementary Fig. 2A through 3B present K-M probabilities of EFS and OS by PET2 response in the subgroups stratified by IPI. Association of PET2 response with survival was strongest in the subgroup of IPI 3-5. PET2 positive patients with IPI 3-5 had significantly worse 2 year EFS (29.3% (CI_95_:19.3–47.3) vs 72.5% (CI_95_:63.9–82.3), *p* < 0.001) and OS (60.1% (CI_95_: 48.7–74.2) vs 84.6 (CI_95_: 77.5–94.2), *p* < 0.001) compared to PET2 negative counterparts.

## Discussion

In two independent prospective cohorts of newly diagnosed DLBCL patients treated with RCHOP-based regimens, we observed that positive PET2 is associated with inferior EFS and OS. There is a strong trend towards inferior OS in MC078E cohort that might not have reached statistical significance due to small sample size (specifically, the small number of deaths). Association of negative PET2 with superior EFS and OS was strongest in the subgroups of IPI 3–5.

One of the limitations of our study include lack of standardized PET results in the MER cohort. PET results were reviewed by electronic medical review and are amenable to subjective bias. However, the fact that survival estimates of MER cohort are quite comparable to the standardized clinical trial cohort of MC078E study speaks to the validity of these results. Another limitation of our data is inability to use most recent version of response criteria. We could not use most recent version of Revised Response Criteria of Malignant Lymphoma as both these cohorts were treated prior to that [19]. Although both discovery and validation cohorts have received different treatments, they can be considered comparable as R2CHOP was proven to have similar response rates, PFS and OS to RCHOP in two randomized controlled clinical trials [14, 15].

In patients treated with RCHOP-14, positive PET2, predicted higher risk of progression but not death [8]. Possible reasons for lack of OS in this study are small sample size and higher rate of false positive PET with RCHOP-14 due to growth factor effect causing stimulation of marrow and spleen uptake [8]. Complete metabolic response, defined as DS 1-3 did not predict PFS or OS in United Kingdom National Cancer Research Institute prospective study of TN-DLBCL [9]. In this study population, comprised of patients treated with both RCHOP-21 and RCHOP-14, response was assessed using 1999 standardized response criteria and DS was assigned by visual assessment of hypermetabolic activity, not SUVmax [9]. In a recent analysis of CALGB 50303 phase 3 trial, positive PET2 by DS did not affect outcomes [10]. In this study, population comprised of 50.9% RCHOP and 49.1% DA-EPOCH-R treated patients, 61% patients had low risk IPI [10]. Small sample size (which is driven by the number of events in this setting) may also have compromised power to detect statistical significance in these studies. A recent meta-analysis of multiple prospective studies showed association of positive PET2 with higher risk of progression [20]. Overall survival was not one of the study outcomes and study population included both RCHOP-21 and RCHOP-14 treated patients [20].

Thus, studies exploring association of PET2 with outcomes in newly diagnosed DLBCL have contradictory results. Our study confirms prognostic impact of PET2 on EFS and OS in two large prospective cohort of newly diagnosed DLBCL in patients. Here are some possible explanations why results of our study differ from prior studies: Prior studies deferred in study treatments. Moreover, patients treated with either RCHOP-14 or DA-EPOCH-R were combined with RCHOP-21 in these studies. Both regimens require neulasta support which can interfere with interpretation of PET scan. Study evaluating impact of PET2 in patients homogenously treated with RCHOP is lacking. Another possible explanation is well-representation of higher risk population in our study, where association of PET2 with outcomes is stronger. Population in some of the prior studies had predominantly low risk patients.

We observed that positive PET2 was associated with higher likelihood of positive EOT PET or progressive disease on EOT PET within the MER cohort. Positive EOT PET has been associated with worse outcomes in newly diagnosed DLBCL treated with RCHOP and is suggestive of primary refractory disease [21–23]. Patients with primary refractory disease are also more likely to be chemorefractory to further line of therapy [4]. Early identification of chemorefractory patients with PET2 can lead to risk-adapted intensification therapy approaches that may improve outcomes in these patients.

Attempts to improve outcomes in newly diagnosed DLBCL with PET2-guided intensification of therapy have been discouraging in chemotherapy era [24, 25]. The PETAL trial was a prospective phase 3 trial that evaluated PET2-guided therapy in newly diagnosed-aggressive NHL treated with RCHOP [24]. PET2 + patients were randomized to have 6 more cycles of RCHOP or a Burkitt protocol [24]. In this study, intensification of frontline treatment with Burkitt protocol failed to improve PFS and OS compared to RCHOP in patients with positive PET2 [24]. A randomized phase 2 trial evaluated consolidation with autologous stem cell transplant (ASCT) in DLBCL patients with positive PET2 after frontline treatment [25]. These patients had similar outcomes to those with PET2- patients who received standard chemoimmunotherapy. But patients in this trial were treated with RCHOP-14 not standard RCHOP [25]. In the era of novel agents such as chimeric antigen therapy and bispecific antibodies, results of our study justify the need for prospective clinical trials evaluating PET2 guided risk adapted frontline strategies of treatment intensification. A phase 2 study of frontline CART cell therapy in PET2 positive patients is currently ongoing and has encouraging results [26]. Results of our study also serve as benchmark for such future clinical trials evaluating PET2 guided risk adapted treatment strategies for newly diagnosed DLBCL.

## Conclusions

Positive PET2 was associated with increased risk of disease progression and death in newly diagnosed DLBCL. Results of our study provide robust evidence of importance of PET2 as an early predictor DLBCL pts at high risk of progression and death in two independent prospective cohorts. PET2-guided risk-adapted strategies incorporating chimeric antigen receptor T-cell therapy and bispecific antibodies may potentially improve outcomes, should be explored in clinical trials and results of our study serve as a benchmark for such studies.

## Supplementary information

Supplementary figures 1,2 and 3
