# Supplementary material for: PET2 response associated with survival in newly diagnosed diffuse large B-cell lymphoma: results of two independent prospective cohorts
Source: Blood Cancer J. 2022 May 3;12(5):78. doi: 10.1038/s41408-022-00649-x (PMC9065135; doi:10.1038/s41408-022-00649-x)
Supplement: Supplementary file 1 — Supplementary figures 1,2 and 3 [file 41408_2022_649_MOESM1_ESM.docx]

Supplementary figure 1A: Association of PET2 response with EFS stratified by age, gender, stage, IPI.

Abbreviations: EFS; event free survival, C2; after 2 cycles of chemotherapy, PET; positron Emission Tomography, PET2; PET scan after 2 cycles of therapy, CI; confidence interval, IPI; international prognostic index.

Supplementary figure 1B: Association of PET2 response with OS stratified by age, gender, stage, IPI.

Abbreviations: OS; Overall survival, C2; after 2 cycles of chemotherapy, PET; positron Emission Tomography, PET2; PET scan after 2 cycles of therapy, CI; confidence interval, IPI; international prognostic index.

Supplementary figure 2A: Association of PET2 response with EFS in the subgroup of low IPI


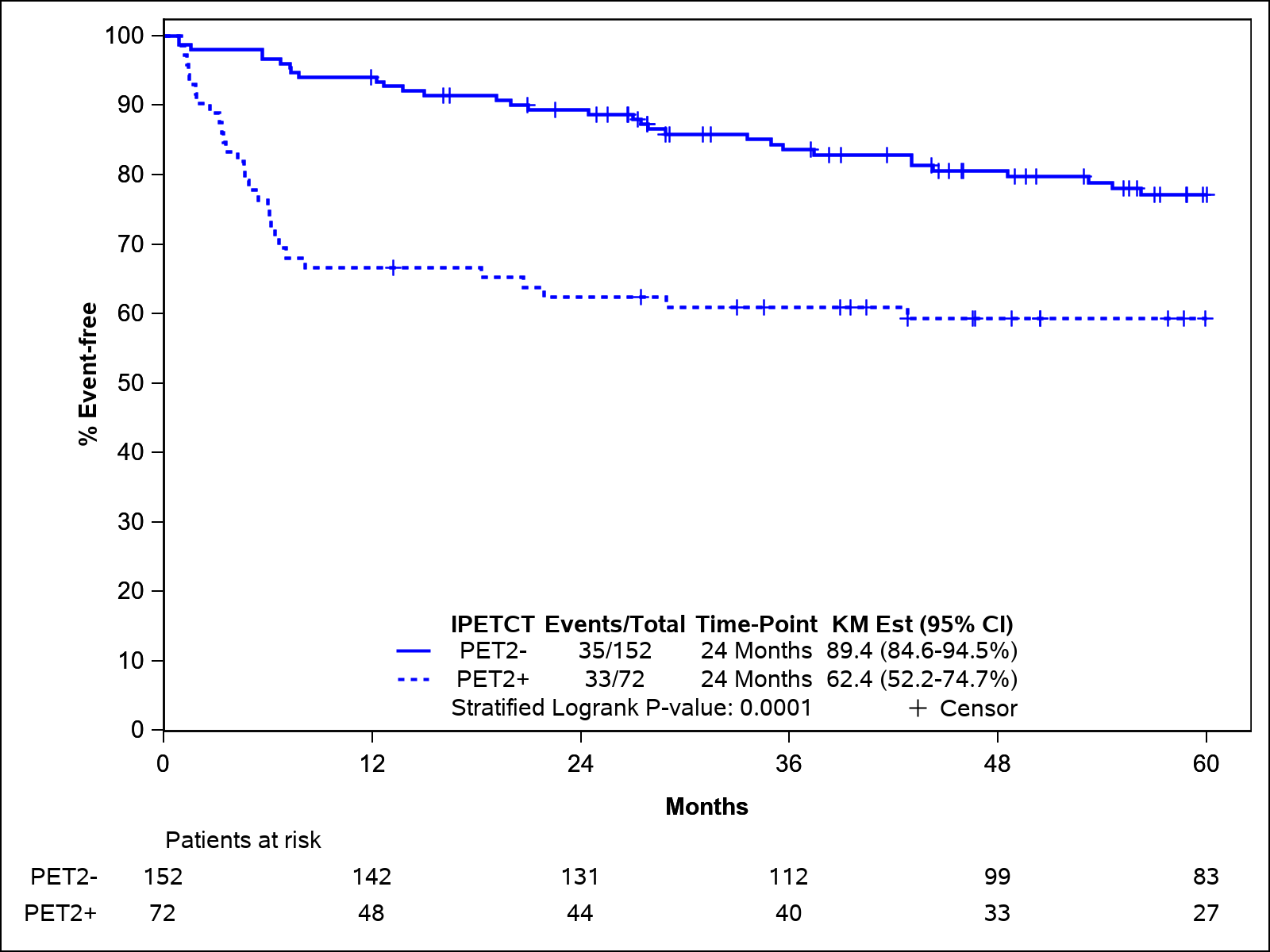


Abbreviations: EFS; event free survival, IPI; international prognostic index, PET2; Positron Emission Tomography (PET) scan after 2 cycles of chemotherapy, PET2-; negative on PET2, PET2+ve; positive on PET2, IPETCT; interim PETCT, KM Est; Kaplan-Meier estimates, CI; confidence interval.

Supplementary figure 2B: Association of PET2 response with EFS in the subgroup of high IPI


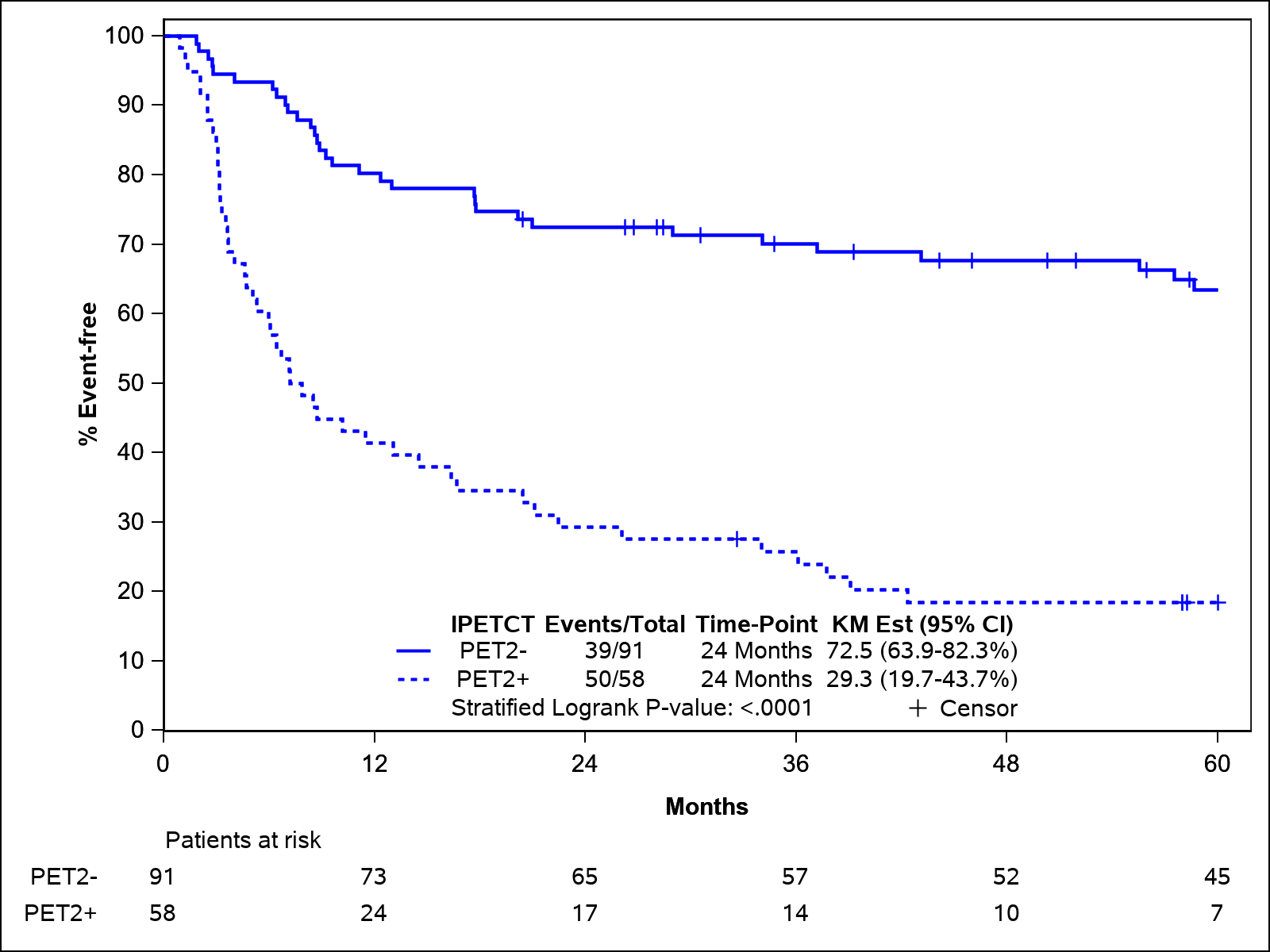


Abbreviations: EFS; Event-free survival, IPI; international prognostic index, PET2; Positron Emission Tomography (PET) scan after 2 cycles of chemotherapy, PET2-; negative on PET2, PET2+ve; positive on PET2, IPETCT; interim PETCT, KM Est; Kaplan-Meier estimates, CI; confidence interval.

Supplementary figure 3A: Association of PET2 response with OS in the subgroup of low IPI


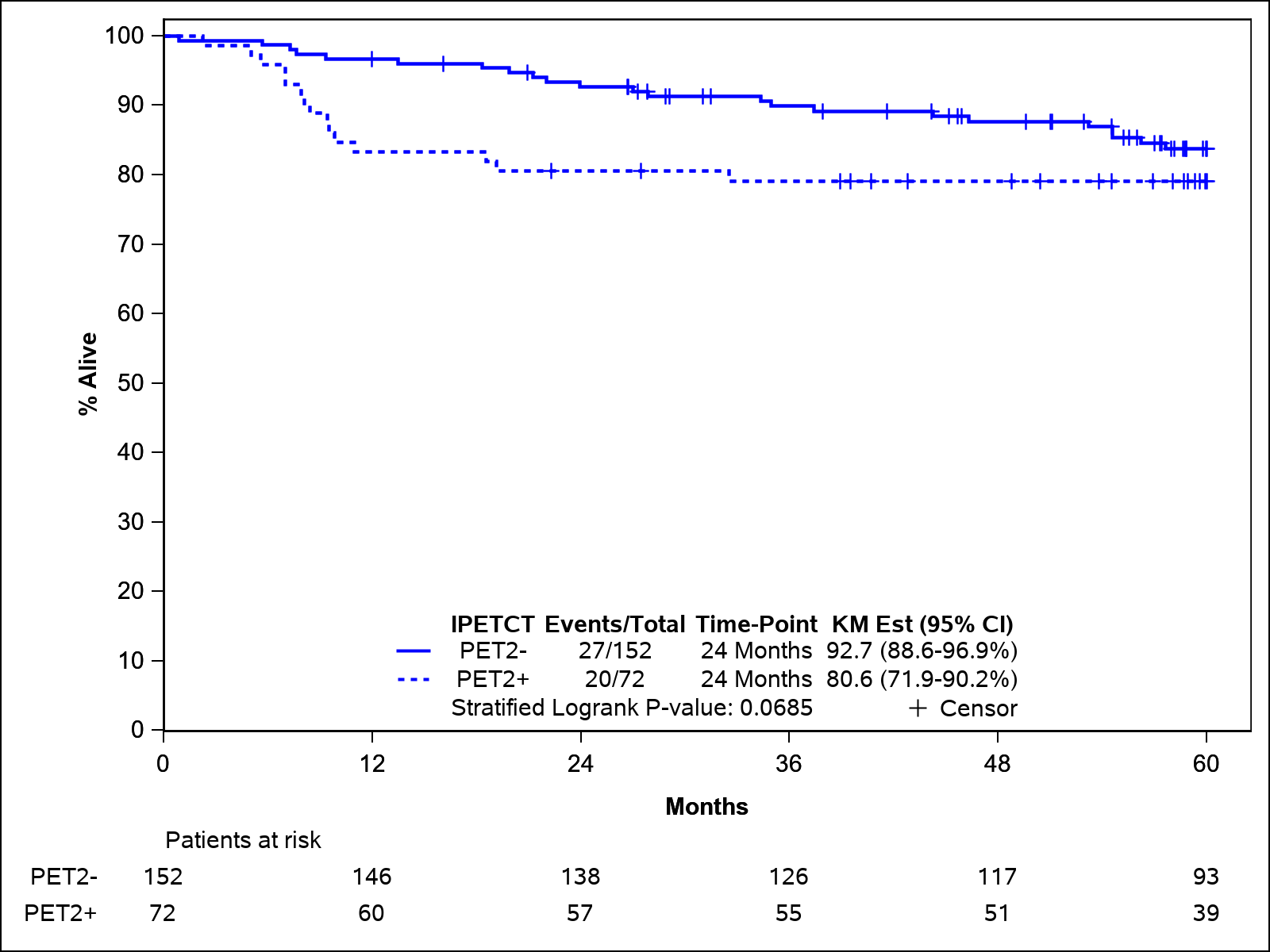


Abbreviations: OS; Overall survival, IPI; international prognostic index, PET2; Positron Emission Tomography (PET) scan after 2 cycles of chemotherapy, PET2-; negative on PET2, PET2+ve; positive on PET2, IPETCT; interim PETCT, KM Est; Kaplan-Meier estimates, CI; confidence interval.

Supplementary figure 3B: Association of PET2 response with OS in the subgroup of high IPI


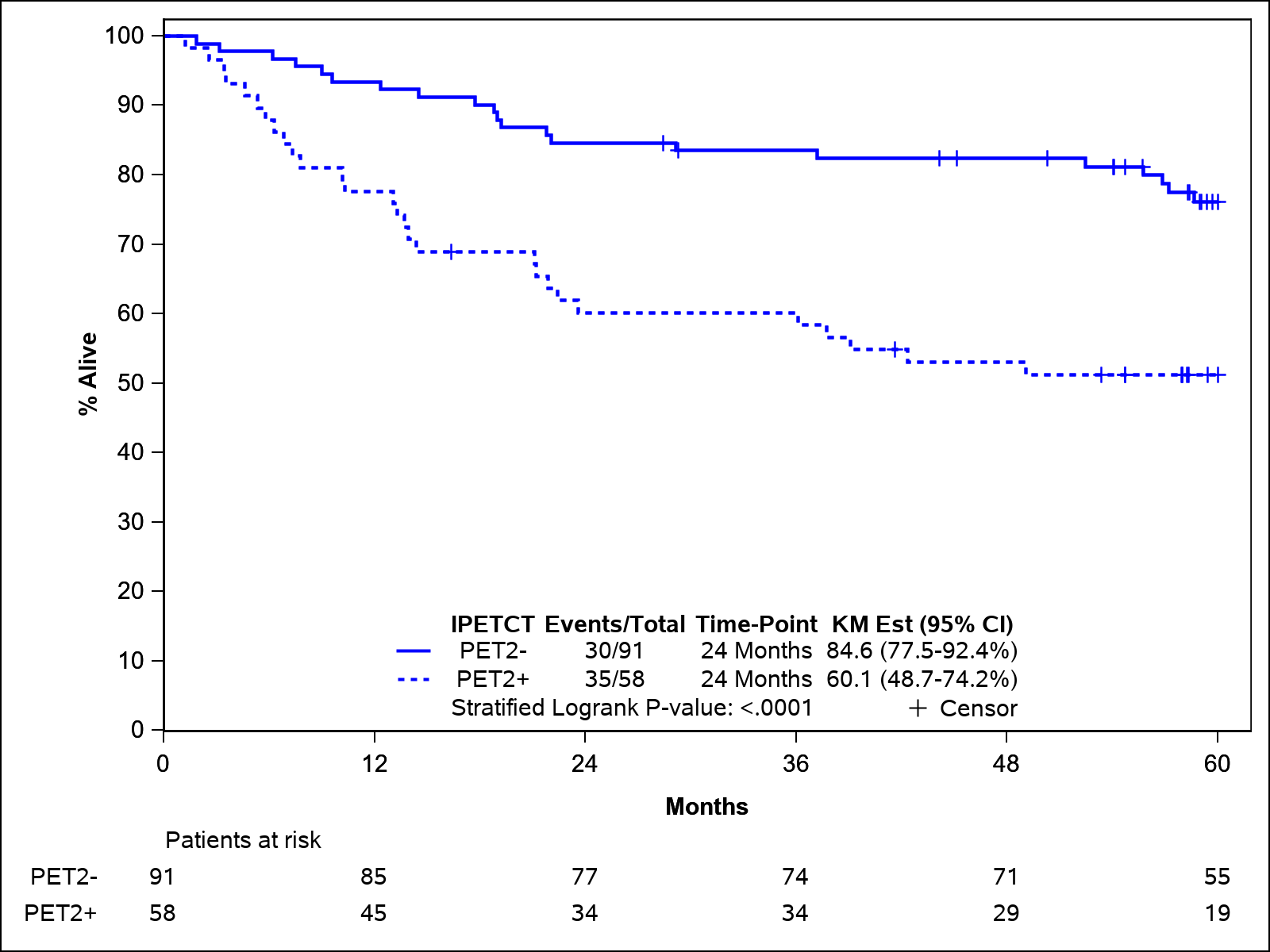


Abbreviations: OS; Overall survival, IPI; international prognostic index, PET2; Positron Emission Tomography (PET) scan after 2 cycles of chemotherapy, PET2-; negative on PET2, PET2+ve; positive on PET2, IPETCT; interim PETCT, KM Est; Kaplan-Meier estimates, CI; confidence interval.
